# Toward a Mathematical Modeling of Diseases’ Impact on Bone Remodeling: Technical Review

**DOI:** 10.3389/fbioe.2020.584198

**Published:** 2020-11-02

**Authors:** Imane Ait Oumghar, Abdelwahed Barkaoui, Patrick Chabrand

**Affiliations:** ^1^Laboratoire des Energies Renouvelables et Matériaux Avancés (LERMA), Université Internationale de Rabat, Rabat-Sala El Jadida, Morocco; ^2^Aix Marseille Univ, CNRS, ISM, Inst Movement Sci, Marseille, France

**Keywords:** bone, bone disease, bone remodeling, mechanobiology, mathematical modeling, drug interventions

## Abstract

A wide variety of bone diseases have hitherto been discovered, such as osteoporosis, Paget’s disease, osteopetrosis, and metastatic bone disease, which are not well defined in terms of changes in biochemical and mechanobiological regulatory factors. Some of these diseases are secondary to other pathologies, including cancer, or to some clinical treatments. To better understand bone behavior and prevent its deterioration, bone biomechanics have been the subject of mathematical modeling that exponentially increased over the last years. These models are becoming increasingly complex. The current paper provides a timely and critical analysis of previously developed bone remodeling mathematical models, particularly those addressing bone diseases. Besides, mechanistic pharmacokinetic/pharmacodynamic (PK/PD) models, which englobe bone disease and its treatment’s effect on bone health. Therefore, the review starts by presenting bone remodeling cycle and mathematical models describing this process, followed by introducing some bone diseases and discussing models of pathological mechanisms affecting bone, and concludes with exhibiting the available bone treatment procedures considered in the PK/PD models.

## Introduction

Bone is continuously renewed through a dynamic biological process, called bone remodeling, which consists of a spatial and temporal coupling of bone resorption and formation phases, allowing to maintain bone calcium homeostasis and preserve its integrity, due to balanced interactions between different bone cells, namely, osteoblasts and osteoclasts. Osteoblasts, which form the bone matrix, are mononucleated cells that derive from mesenchymal stem cells (MSCs). These MSCs are multipotent stromal cells able to differentiate into a multitude of different cells, owing to their gene expression program (e.g., osteoblasts, fibroblasts, adipocytes, and chondrocytes; Grigoriadis et al., 1988; Yamaguchi and Kahn, 1991). On the other hand, osteoclasts, which resorb the bone matrix, are multinucleated cells that derive from hematopoietic stem cells. As a result of preosteoclast fusion, the created active osteoclasts become multinucleated where the nuclei’s number can vary between 4 and 20 nuclei (Udagawa et al., 1990). However, imbalanced interactions between bone cells lead to impaired remodeling process, which results in several metabolic bone diseases, mainly osteoporosis and Paget’s disease of bone (PDB).

Osteoporosis is a biochemical defect characterized by a decrease of bone mass, as well as a deterioration and an alteration of bone tissue microarchitecture, which leads to increased fracture risk and structure damage of the bone. Yet osteoporosis may be more pronounced in the case of unfavorable conditions, such as genetics, daily diets, hormonal secretion, or smoking history (Barry et al., 2012; Yedavally-Yellayi et al., 2018). On the other hand, PDB is a chronic bone disease, classified as the second most common bone disease after osteoporosis. It consists of a focal disorder of bone remodeling, which leads to persistent changes in single or multiple bone’s shape and size. The region or regions affected by PDB undergo excessive bone remodeling, characterized by increased bone resorption followed by disorganized and excessive bone formation (Sabharwal et al., 2014). Paget’s disease mainly affect elderly people, with a preponderance of 1–5% at the age of 50 years (Appelman-Dijkstra and Papapoulos, 2018).

In addition to the above-mentioned factors, cancer, particularly breast cancer (BC), prostate cancer (PC), and multiple myeloma (MM), is considered as one of the main factors leading to the occurrence of several bone diseases. Indeed, BC is one of the most widespread diseases among women, especially after menopause; as by 2025, 1.1 billion postmenopausal women are estimated to develop this type of cancer (Labrie, 2015), whereas PC is known to be the most diagnosed non-cutaneous cancer in the world for men, with 0.9 million diagnosed cases per year (Parkin et al., 2005), affecting frequently elderly people. As regards MM, this disease, also called plasma cell myeloma, is a blood cancer characterized by an invasive growth of B-lymphocytes during their final stage of differentiation leading to malignant plasma cells (MPCs). It represents the second most frequent hematological malignancy, with a prevalence of 28–37% in elderly people (Palumbo and Rajkumar, 2009).

The focus of the current review is on presenting the biological and pathological factors involved in the development of bone diseases. Knowing that mathematical models have shown a great potential in mimicking the spatial and temporal evolution of bone cells during bone remodeling cycle and clarifying several complicated biological interactions, this paper presents the different methods used to describe the pathophysiological mechanisms of a diseased bone. Based on our understanding, the reviewed studies are discussed from a critical point of view to (i) facilitate the comprehension of the biological factors involved within bone remodeling, (ii) give an overview of the methods used to incorporate the effects of bone diseases into a biological model, and (iii) discuss the strategies adopted to study the drug treatments’ potential in limiting some bone diseases.

## Bone Remodeling

### Experimental Observation

The activity of cells leading to bone turnover was identified for the first time by Frost (1969) as a bone multicellular unit (BMU). BMU is a key operator of the remodeling process as it occurs in all the skeletal compartments to ensure the renewing of spatial regions. It involves osteoblast and osteoclast lineage cells, in addition to blood vessels associated with the connective tissue once it is fully developed. The change in the spatial/temporal coordination of bone cell activities affects bone mass quantity and leads to several bone pathologies. Osteoblast and osteoclast behaviors are the predominant mediators of bone turnover, and their activities are under the control of a number of biological factors.

**FIGURE 1:**
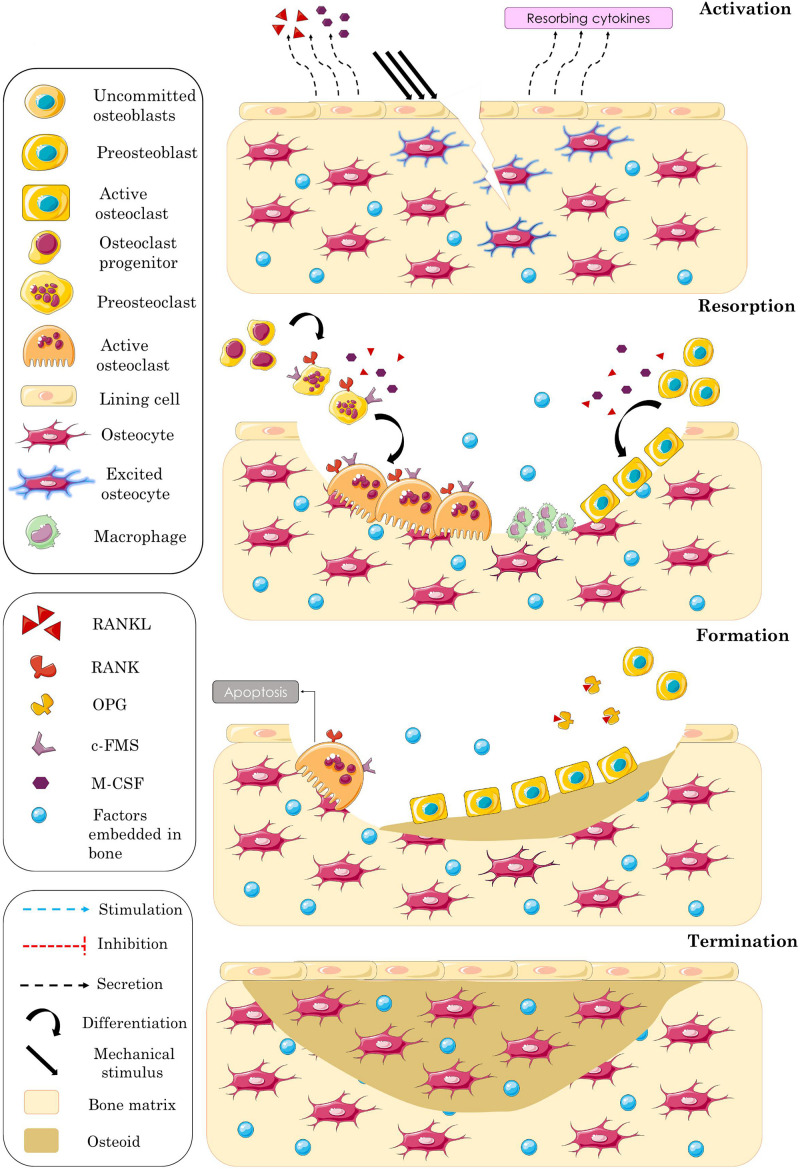
Schematic representation of the different overlapping phases of bone remodeling process. Activation phase: excitation of embedded osteocytes leading to recruit resorbing cells by means of biochemical factors. Resorption phase: bone matrix resorption accompanied by osteoblasts’ recruitment. Formation phase: blocked osteoclasts’ activity accompanied by progressive osteoid synthesis. Termination phase: mineralization of the formed bone matrix and completion of the remodeling process.

Bone remodeling consists of a sequence of four events allowing to maintain bone strength, notably activation, resorption, formation, and termination (Figure 1).

#### Activation

The initiation of bone remodeling is governed by two types of stimuli factors: (i) the biochemical factors established by the variation of hormone secretion and (ii) the mechanical ones established by the mechanical loading applied to bone. The mechanical stimulus is detected by osteocytes embedded in mature bone matrix and traduced to biochemical information [e.g., sclerostin (SCLR) and nitric oxide (NO)] that activates the BMU. Bone lining cells, which are quiescent osteoblasts, receive these biochemical signals and start to release resorbing cytokines, such as the receptor activator of NFκB ligand (RANKL) and the macrophage colony-stimulating factor (M-CSF), that stimulate osteoclast differentiation and activation.

#### Resorption

While binding to its receptor c-FMS, M-CSF stimulates the expression of the receptor activator of NFκB (RANK) by preosteoclasts, and the binding of RANKL to its RANK receptor promotes the osteoclastogenesis. Osteoclast regulation is mediated by many other factors like prostaglandin E2 (PGE_2_), parathyroid hormone (PTH), and the active form of vitamin D3 [1.25(OH)_2_D_3_], which stimulate osteoblasts’ release of factors influencing osteoclast activity (Lutter et al., 2016). Likewise, there are inhibiting factors of osteoclastogenesis such as osteoprotegerin (OPG), which plays an important role in bone remodeling as a soluble decoy receptor for RANKL (Simonet et al., 1997). This biochemical factor is highly expressed by osteoblast lineage cells and has the ability to prevent the formation of the RANK/RANKL complex as it binds RANKL with approximately 500-fold higher affinity than RANK (Nelson et al., 2012; Infante et al., 2019). After activation, osteoclasts adhere to bone surface and attach into it by means of integrins that bind the amino acid sequences of the bone matrix proteins (Davies et al., 1989). Subsequently, they start secreting hydrogen ions and acid phosphatases to acidify the mineral compartment and releasing enzymes to resorb the old organic matrix (Väänänen et al., 2000; Väänänen, 2005). Several cytokines and systemic hormones are involved in old bone removal, such as PTH (Teti et al., 1991), interleukin-1 (IL-1) (Xu et al., 1996), and insulin-like growth factor-1 (IGF-1) (Hou et al., 1997). At the end of the resorption phase, osteoclasts undergo apoptosis (Teitelbaum, 2000), and macrophages clean the surface from the remaining debris. Then, osteoblasts migrate into the bone lacunae, and the formation phase is initiated (Rucci, 2008).

#### Formation

During this third remodeling phase, osteoblasts are recruited to produce osteoid by synthetizing collagen, and their activity is regulated by several growth factors, such as IGF (Canalis et al., 1993a), transforming growth factor β (TGF_β_) (Canalis et al., 1993b), and bone morphogenetic protein (BMP) (Chen et al., 2004). These growth factors are initially incorporated into the old mineralized bone matrix and released after its resorption. During matrix deposition, the osteoid is gradually mineralized, and some osteoblasts are trapped within the newly formed bone matrix and differentiate into osteocytes that are interconnected with each other, forming a 3D communication network. At the end of the formation phase, the remaining osteoblasts either differentiate into lining cells, creating a thin layer covering the bone surface, or undergo apoptosis (Hadjidakis and Androulakis, 2006).

#### Termination

In physiological conditions, where the bone cavity is nearly filled at the end of formation, osteoblasts activity is slowed, and the BMU’s recruitment gets smaller. According to some researchers, this stage is reached when osteocytes secrete inhibitory factors that repress bone formation (Parra-Torres et al., 2013) or when they stop expressing the biochemical factors that activate the other bone cells, namely, SCLR (Raggatt and Partridge, 2010). Therefore, the real mechanism behind this phenomenon is not clearly identified.

Understanding the remodeling mechanism and cell behavior during physiological bone metabolism is a crucial step to build a mathematical model describing this vital process and to discern the various pathologies related to the coordination between the resorption and formation activities.

### Mathematical Models of Bone Remodeling

Many mathematical models have been developed to schematize and predict bone remodeling behavior over time (Pivonka and Komarova, 2010). Therefore, this method becomes a promising tool in predicting bone quality changes based on the bone response to specified biological and mechanical conditions. In order to understand this biological process of bone, several approaches have been suggested. The phenomenological approach, for instance, has been formulated by Cowin and Hegedus (1976) based on the continuum mechanics theory. This paper presented the first continuum mathematical formulation of bone remodeling, where the salient biological and physical features of the process were taken into consideration. Yet, the model was focusing more on the mechanical aspect than the cellular interactions during the process. In this study, bone matrix was defined as porous elastic structure whose mass varies mainly depending on the local strain. For more simplification, other researchers have considered bone as isotropic material in their studies (Huiskes et al., 1987; Weinans, 1989; Weinans et al., 1992). Despite the non-validity of this assumption, the isotropic models are, nowadays, the most used in the literature. Indeed, while investigating bone mechanical performance or the risk of its fracture, researchers are more interested in the cortical bone compartment, which is a transversely isotropic material (Dong and Guo, 2004), than the trabecular bone compartment. The second bone remodeling model that has been proposed in the literature is presented in Beaupré et al. (1990), where bone has been taken as an isotropic material whose apparent density depends on strain energy density.

Other model types have also been proposed in the literature, where the resorption and formation of bone matrix at the cellular level are considered. These models are also extremely important in the history of mathematical modeling of bone. Several studies has been done in this respect such as Frost (1969) and Turner (1991), were the BMU notion and the bone cell activity relationship with mechanical stimulus has been described. These models facilitated the understanding of bone cells roles. Besides, they linked the physiological phenomena occurring at the cellular level with the changes of bone mechanical properties.

For the last two decades, advanced models, which include cell population within the remodeling process, have been widely developed, as they are clear and allow the possibility to be modified. There is a variety of ways these models are conceived: (i) the dynamic models based on a system of ordinary differential equations (ODEs), which are temporal-only models, with no consideration of spatial effects of BMUs and which represent bone cell populations (Komarova et al., 2003; Lemaire et al., 2004; Pivonka et al., 2008); (ii) the continuum models based on partial differential equations (PDEs), which are not widely used (Ryser et al., 2009; Buenzli et al., 2011); and (iii) the discrete models (van Oers et al., 2008; Buenzli et al., 2012).

Recently, mechano-chemo-biological models, which derive from the ODE models previously mentioned, have appeared. Very few articles have considered this type of models in their investigations (Klika et al., 2013; Avval et al., 2014; Lerebours et al., 2016; Pastrama et al., 2018; Martin et al., 2019; Ashrafi et al., 2020). Nevertheless, we think that they are very promising as they englobe each of mechanical, biological, and biochemical features of bone. Indeed, these models take into consideration the mechanotransduction property of osteocytes and show its effect on bone cells response.

In the current paper, we are focusing on the ODE models. The model of Komarova et al. (2003) represents the gold standard of the ODE models, depicting bone cell behavior throughout the turnover process. Autocrine and paracrine interactions involving both of the considered cell populations are taken into account such that all processes are modeled as power laws. However, it was pointed out that the proposed model is more sensitive to the osteoclast autocrine regulation, that is, to the influence of TGF_β_, while the change in osteoblasts is very restricted, which requires imposing a higher number of osteoblasts in each remodeling cycle. Besides, a stabilization problem of the steady state was detected by Zumsande et al. (2011). The effects of the biological factors addressed in this model cannot be distinguished, and the different phases of cell maturation cannot be examined. Still, the model provided an important step forward in bone biology. A more detailed model was formulated by Pivonka et al. (2008), incorporating more controlling factors using Michaelis–Menten kinetics and calculating the evolution of the concentrations of three types of bone cells that are preosteoblasts, active osteoblasts, and active osteoclasts. Indeed, the RANKL/OPG ratio varies at different cell maturation stages (Gori et al., 2000; Thomas et al., 2001), and osteoblast differentiation at early stages is only activated by TGF_β_; otherwise, the differentiation is repressed (Janssens et al., 2005). Therefore, it is necessary to consider osteoblast lineage cells before activation. A model described in Zumsande et al. (2011) was found to stimulate the sensitivity of bone volume changes to the differentiation rates, where RANKL is considered to be only expressed by preosteoblasts, while OPG is expressed by active osteoblasts. The results of this study showed that taking preosteoblasts into account plays an important role in stabilizing the dynamic system, unlike the forgoing model of Komarova et al. (2003), where the stability of the steady state was related to the amount of OPG that needs to dominate that of RANKL.

The major interest of researchers, by developing all these models, is to improve the understanding of bone biology and to predict and prevent bone defects, which cannot be assessed *in vivo*. Thus, several mathematical models have been developed based on ODEs, seeking the description of the influence of bone diseases on its cell behavior and mass density. Indeed, according to the targeted aim, researchers elaborate particular experimentations to understand the assumption they will base their work on and to feed their models with accurate parameters. These parameters are enhancing the capacity of the model in mimicking the targeted phenomenon. Thus, the model results could be validated based on the elaborated experimentation. For these reasons, we can conclude that there is no perfect model and each model has its particularities.

## Bone Diseases

### Experimental Observations

#### Osteoporosis

Etiologically, osteoporosis is subdivided into two categories: (i) primary osteoporosis, which is associated with sex, age, and hormone deficiency (e.g., reduced estrogen in postmenopausal women); and (ii) secondary osteoporosis, which results from the onset of some diseases or from undergoing a medical treatment that stimulates osteoclast activity (Bonnick et al., 2010). Osteoporotic fractures generally occur in the spine, hip, and wrist and can be detected in the case of a low value of bone mineral density (BMD) at the fractured site (Barry et al., 2012). Indeed, BMD value is the most used parameter to predict fracture risk in adults. According to the World Health Organization (WHO), bone state can be classified into four groups (Table 1), based on the value of the T-score, which represents the number of standard deviation (SD) between the BMD value of a specific patient and the average value in an adult of the same sex.

**TABLE 1:** T-score-associated bone quality according to WHO diagnostic criteria for osteoporosis (Tu et al., 2018).

**Interpretation**	**T-score**
Normal	−1.0 and higher
Osteopenia	−1.0 to 2.5
Osteoporosis	−2.5 and lower
Severe osteoporosis	−2.5 and lower with one or more fragility fractures

In fact, osteoporosis is a silent disease and becomes more pronounced with age, which requires a regular screening of the BMD. This screening is recommended for women above the age of 65 and for subjects with a fracture history above the age of 50 (Barr et al., 2010; Cosman et al., 2014; Camacho et al., 2016; Curry et al., 2018).

#### Paget’s Disease

The clinical presentation of PDB is highly variable in that some patients are asymptomatic or have few symptoms, whereas others develop several complications, such as bone pain, fracture, deformity, and deafness. The real etiology of PDB is still unknown. Yet it was found that up to 40% of patients with family history of PDB have a mutation in *SQSTM1* gene, with a p62 protein involved in osteoclast regulation (Ralston and Layfield, 2012). This suggests that PDB is likely caused by a genetic slow paramyxoviral infection. The affected bone areas show an increase in the number and size of multinucleated osteoclasts during bone remodeling. The subsequent excessive resorption leads to the increase in osteoblast recruitment and, thereby, to an excessive bone formation rate. Besides, PDB results in a disorganized architecture and a significant fragility, because of the higher remodeling rate compared with that in a healthy bone, as well as the dysregulation in the coordination between osteoclast and osteoblast activities (Appelman-Dijkstra and Papapoulos, 2018). Over the recent years, many countries have registered a decrease in PDB prevalence, and this is likely related to the change in daily diets and environmental factors influencing the manifestation of this pathology, in addition to the high exposure of bone to mechanical loading (Ralston and Layfield, 2012).

In fact, PDB mainly affects the pelvis, the spine, the femur, and the skull. Since an increase in the activity of the alkaline phosphatase (ALP) reflects an increase in osteoblast activity, PDB can be detected by analyzing blood samples to quantify the ALP serum level. It can also be detected using medical bone scanning to directly observe the change in the thickness of bone matrix (Kravets, 2018). In the most advanced stages, PDB may lead to other serious complications (Table 2).

**TABLE 2:** Complications and clinical manifestation of Paget’s disease of bone (Ralston, 2013).

Musculoskeletal	Bone pain - bowing of long bones - enlarged skull – osteoarthritis of joints adjacent to pagetic lesions – bone fractures – sarcoma
	– giant cell tumors
Neurological	Hearing loss – platybasia – spinal stenosis – vascular steal syndromes – cranial nerve deficits (rare)
Cardiovascular	High output heart failure – aortic stenosis – endocardial calcifications
Genitourinary	Nephrolithiasis
Metabolic	Hypercalcemia (in some patients) – immobilization hypercalciuria – hyperuricemia

#### Cancer-Associated Bone Diseases

Cancer cells have the ability of impacting bone turnover and cause its dysregulation through several complex biological factors (Roodman, 2004), which is the case of BC, PC, and MM.

#### Breast Cancer-Associated Bone Disease

Postmenopausal women are the closest to develop BC because of many reasons, such as the decrease in estrogens and progesterone at the fifth decade, which increases the risk of developing osteoporosis. Once they arrived at the bone marrow, epithelial tumor cells start interacting with bone cells by secreting different types of cytokines such as the IL group IL-1, IL-6, IL-8, IL-11, M-CSF, BMP, dickkopf-related protein-1 (DKK-1), Activin A, PGE2, and the PTH-related peptide (PTHrP) (Clézardin, 2011). Each cytokine plays a different role, but all of them inhibit bone formation and stimulate bone resorption. Particularly, BC cells promote the production of RANKL by osteoblasts and inhibit their production of OPG (Infante et al., 2019).

#### Prostate Cancer-Associated Bone Diseases

Similarly to BC, PC affects elderly people who already usually suffer from age-related bone loss (Brown et al., 2010). While progressing, this type of cancer cells preferentially metastasizes bone tissue, instead of other types of tissues in the human body. Thus, roughly 90% of men with advanced PC can have bone micrometastasis (Bubendorf et al., 2000). Unlike the majority of cancers that induce bone osteolytic lesions, PC is associated with osteoblastic lesions (Logothetis and Lin, 2005). When PC cells arrive at the bone microenvironment, they entirely disrupt the balanced interactions between bone cells. According to Farhat et al. (2017), PC cells produce a high amount of wingless-int (Wnt), which makes them the major dysregulators of bone remodeling process. They also produce DKK-1 (Hall et al., 2008) that regulates Wnt signaling and suppresses osteoblastogenesis (Krishnan et al., 2006; Heath et al., 2009) and PTHrP (Asadi et al., 1996) that regulates the communication between bone cells and PC cells (Liao et al., 2008). When adapted to bone microenvironment, PC cells start secreting the prostate-specific antigen (PSA), which inhibits PTHrP production (Cramer et al., 1996). Besides, Wnt stimulates bone formation by increasing the number of osteoblasts. This causes an increase in the production rate of RANKL (Boyce and Xing, 2007; Yavropoulou and Yovos, 2007), which stimulates the formation of osteoclasts. The subsequent increase in the resorption rate is associated with the increase in the amount of the latent TGF_β_ (LTGF_β_), leading to the stimulation of PC proliferation (Langdahl et al., 1997).

#### Multiple Myeloma-Associated Bone Diseases

Symptomatic and asymptomatic MMs are highly related to end-organ damage. Myeloma bone disease (MBD) affects approximately 60% of patients (Coleman, 1997), and its development is associated with bone fractures, pain, hypercalcemia, and the compression of the spinal cord (Coleman, 1997; Terpos et al., 2005). Some of these symptoms are mainly related to the disruption of bone remodeling mechanism. Unlike the other types of cancer that stimulate the activities of osteoblasts and osteoclasts, MM stimulates osteoclastogenesis and inhibits osteoblastogenesis, which increases the resorption rate and decreases the formation rate. Indeed, MPCs interact with bone marrow stromal cells (BMSCs) and extracellular matrix through molecule adhesion. These interactions stimulate the production of IL-6, which promotes the survival of malignant plasma (Walker et al., 2014). The increase in MM spread enhances the expression of signaling factors promoting osteoclast differentiation and functioning, namely, RANKL, IL-3, IL-6, and IL-7. Besides, the MM-derived exosomes stimulate the migration, the survival, and the differentiation of osteoclasts (Wu et al., 2003). On the other hand, cancer cells inhibit the differentiation and the proliferation of osteoblasts by releasing Wnt antagonists, such as DKK-1. Many other factors, such as IL-2, IL-6, IL-7, and vascular endothelial growth factor (VEGF) are involved in the development of MBD by affecting the balance of the RANKL/OPG complex and causing the inflammation of macrophages (Hameed et al., 2014).

### Mathematical Models Treating Bone Diseases

#### Bone Remodeling Models Considering Osteoporosis and Paget’s Bone Disease

A theoretical model was developed by Lemaire et al. (2004) to study the effects of osteoporosis on the biochemical network controlling the remodeling process. The authors considered that osteoporosis is caused by estrogen deficiency in postmenopausal women, 1.25(OH)_2_D_3_ deficiency, and glucocorticoid excess. The incorporation of the effect of osteoporosis into the model is explained in Table 4, and the effect of each change in bone cells is presented in Table 3. The results obtained from these models show a high correspondence with experimental and clinical observations. Therefore, the changes made on this mathematical model should be taken into consideration in future research according to the type of osteoporosis assessed.

**TABLE 3:** Effect of osteoporosis on bone turnover and cell behavior in the case of different disorders: estrogen and vitamin D deficiency and glucocorticoid excess (Lemaire et al., 2004).

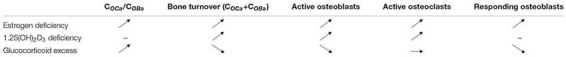

*C_*oca*_ and C_*oBa*_ represent the concentration of active osteoclasts and active osteoblasts respectively.*

Likewise, Pivonka et al. (2013) treated osteoporosis and suggested a bone remodeling mathematical model considering bone geometrical regulation and bone surface availability. Osteoporosis is caused by PTH excess (Table 4) and is related to the increase in bone porosity. Hence, the vascular porosity in this study was calculated based on the results of the behavior of bone cells in an osteoporotic bone and used to estimate the changes in the specific surface based on the work of Martin (1972). The resulting specific surface was integrated into the remodeling model; thus, for each calculated specific surface, the behavior of the cell populations was altered. According to the findings, this research shows that the geometrical regulation of BMU may be implicated in the development of bone porosity, while the specific surface does not have any significant influence on this last’s evolution.

**TABLE 4:** Methods adopted to incorporate osteoporosis, PDB, PC, and MM in mathematical models.

**Disease**	**Cause**	**Changed parameters**	**Method**	**References**
Osteoporosis	Menopause	Minimal rate of OPG production	Decreasing the value until C_*OCa*_/C_*OBa*_, in the steady state, reaches 5 (reflecting the osteoporotic bone)	Lemaire et al., 2004
	1.25(OH)_2_D_3_ deficiency	PTH production rate	Increasing the value until it reaches 3,765 pM/day	
	Glucocorticoid excess	Differentiation rates of osteoblast progenitors	Reduced to reach 1.7 × 10^–4^ pM/day in order to simulate the biological dysregulation	
	-	PTH concentration	Increasing its value as PTH perturbs the homeostatic steady state of bone cells by inducing RANKL/OPG ratio rise	Pivonka et al., 2013
PDB	-	Autocrine parameters—normalized activity of resorption and formation	- Increasing the formation rates of bone cells - Increasing the value of the autocrine parameters - Increasing bone resorption activity parameter and reducing the formation one.	Komarova et al., 2003
Malignant bone	Prostate cancer	- Activation function of preosteoblast differentiation - TGF_β_ concentration - OPG concentration - PTH concentration	- Adding the effect of Wnt as stimulator of preosteoblast differentiation - Wnt production by PC cells is repressed by DKK-1 - Adding an activation function of TGF_β_ activation mediated by PSA binding to its receptor - Considering PTHrP concentration in the calculation of repression function controlling OPG production	Farhat et al., 2017
	Multiple myeloma	- Autocrine and paracrine parameters	- Autocrine and paracrine parameters depend on the tumor evolution; while tumor’s density increases, they increase for osteoclasts’ case and decrease for osteoblasts’ case	Ayati et al., 2010
		- RANKL concentration	- Effective carrying capacity on preosteoblast equation, which enters into RANKL concentration calculation, depends on the activation function mediated by PTH and IL-6 binding to their receptors. - IL-6 production by uncommitted osteoblasts is controlled by the activation function mediated by VLA4 and TGF_β_ binding to their receptors.	Wang et al., 2011
		- Differentiation from preosteoblasts to active osteoblast term - Apoptosis of active osteoblasts term -RANKL concentration	- Adding a repression function of preosteoblast differentiation mediated by VCAM1 binding to its receptor. - Adding an activation function of preosteoblasts differentiation mediated to VCAM1 binding to its receptor. - Effective RANKL concentration controlled by IL-6 activation function. - IL-6 production by uncommitted osteoblasts is controlled by the activation function mediated by VLA4 and TGF_β_ binding to their receptors. - VLA4 production depends on MM cell concentration. - MM cell proliferation is repressed by the repression function mediated by SLRP binding to its receptor.	Ji et al., 2014

In the research paper of Komarova et al. (2003), the authors found that their model established an unstable behavior of bone cells, which is similar to the behavior in Paget’s disease. By imposing specific conditions leading to unstable oscillation (Table 4), the authors observed an increase in the number of osteoblasts and osteoclasts over time. Therefore, bone mass increases due to the alteration of increased bone resorption followed by increased bone formation. The developed model shows a higher sensitivity to the effectiveness of the autocrine regulation of osteoclasts and osteoblasts, compared with the normalized activity of bone resorption and formation. Although the results are consistent with the characteristics observed in Paget’s disease, a clearer representation of the disease is required in the future research. Actually, the different factors inducing this type of diseases should be implemented in such a way that their real effects on bone remodeling can be detected. But in this model, the autocrine and paracrine parameters did not reflect the effect of a single biological factor.

#### Bone Remodeling Considering Cancer Diseases

Different types of cancer have been reported to cause bone metastasis. Therefore, it is necessary to investigate their influence on the remodeling process. The paper of Garzón-Alvarado (2012) provides a description of a standard bone remodeling mathematical model, which can be used to show the effects of cancer on bone at a cellular level. In fact, there are two types of bone disorders caused by cancer: (i) osteosclerosis, which is an increase in bone mass; and (ii) osteolysis, which is a decrease in bone mass. In this model, the type of the pathogenesis is determined based on a differential equation calculating bone mass proportion variation that depends on normal bone cells and those affected by cancer.

In order to recognize the type of bone metastasis, an equation was included to determine the effect of the tumor growth depending on the maximum normal number of the ratio of osteoblasts to osteoclasts. After cancer spread, the evolution of the secondary tumor growth was controlled by using specific concentrations of TGF_β_. On the other hand, the concentrations of PTHrP and IGF were modeled through differential equations depending on the number of cancer cells, in order to influence the behavior of bone cells according to the type of the cancer (i.e., IGF representing osteosclerosis and PTHrP representing osteolysis). The results provided were nearly similar to those of previous experimental studies. However, as the model parameters are based on numerical experimentation, the results may not represent the reality.

#### Prostate Cancer Disease

**FIGURE 2:**
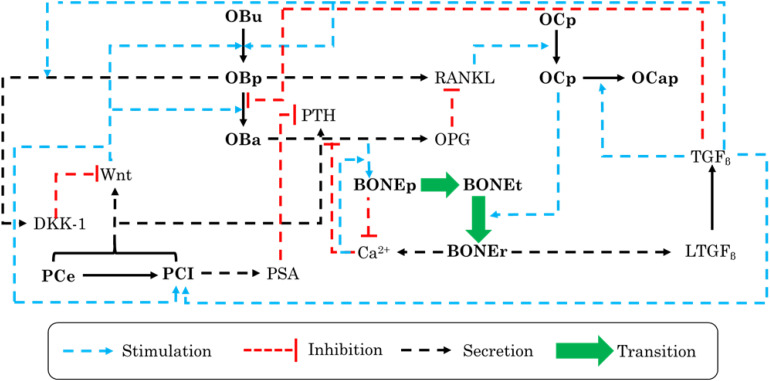
Schematic illustration of the biochemical interactions and feedback loops within a bone remodeling process in the presence of prostate cancer (PC) cells that were adopted in the mathematical model. OBu, uncommitted osteoblasts; OBp, preosteoblasts; OBa, active osteoblasts; OCp, preosteoclasts; OCa; active osteoclasts; OCap, apoptotic osteoclasts; PCe, early PC cells; PCI, late PC cells; BONEt, total bone; BONEp, bone production; and BONEr, bone resorption.

A model developed by Farhat et al. (2017), based on the work of Wang et al. (2011), represents the interactivity of PC cells with the bone microenvironment and properly established the impact of PC growth on the remodeling process, using a mathematical model, where each influencing factor has been simply explained (Table 4). In order to interlink the dynamic interplay between PC cells and bone cells, the authors included many biological factors that are sensitive to PC cells (Figure 2).

The main changes included in the mathematical model and the main conclusions of the study are presented in Table 5. Based on the results, authors have discovered the existence of two osteogenic states, low and high. However, this cannot be validated by experimentation unless a continuous measurement of some interacting biochemical factor levels, over the entire course of the disease, is carried out.

**TABLE 5:** Main changes adopted in the model of Farhat et al. (2017) and the main result.

**Main changes**	**Main result**
- Uncommitted osteoblast differentiation into preosteoblasts is governed by each of TGF_β_ and Wnt. - Active osteoclasts’ apoptosis varied from a base rate noted *α_*oca*_*_1_, and then, it follows TGF_β_ ’s concentration. - Bone formation is controlled by calcium concentration and not only by bone cell activity.	There are two levels of osteogenic states, the low, which depends on TGF_β_ activation by prostate cancer cells, and the high, which is related to the Wnt existence.

*Wnt, wingless-int.*

To our knowledge, the effect of BC on the remodeling process remains neglected in such kind of research. Thus, the article of Farhat et al. (2017) can represent a basic platform to create a model showing the interaction between BC cells and bone cells. Besides, a computational model can also be developed based on the spatial dimension, such as in the work of Araujo et al. (2014).

#### Multiple Myeloma Cancer Disease

Similar to PC, myeloma-associated bone remodeling has also been studied through mathematical models. The model developed by Ayati et al. (2010) showed the influence of tumor growth on the remodeling process. The authors adopted the model of Komarova et al. (2003) and added a tumor function that disrupted the normal oscillation of the number of bone cells during remodeling, by influencing the autocrine and paracrine parameters (Table 4). This function represented the evolution of the tumor density, which has the form of Gompertz function and depends on the maximum size and the growth constant of the tumor. The results of the developed model reflected an important representation of MBD, where the numbers of osteoclasts and osteoblasts damped oscillations, did not converge to steady state, and induced a progressive decrease in bone mass. Nevertheless, we presume that, in this work, more attention should be addressed to the biological impact. The parameters, either of the remodeling process or of the tumor growth, were not explicitly explained regarding their biological meaning. Thus, it would be hard to specify the factors directly controlling the progression of the MM cells and the behavior of bone mass.

**FIGURE 3:**
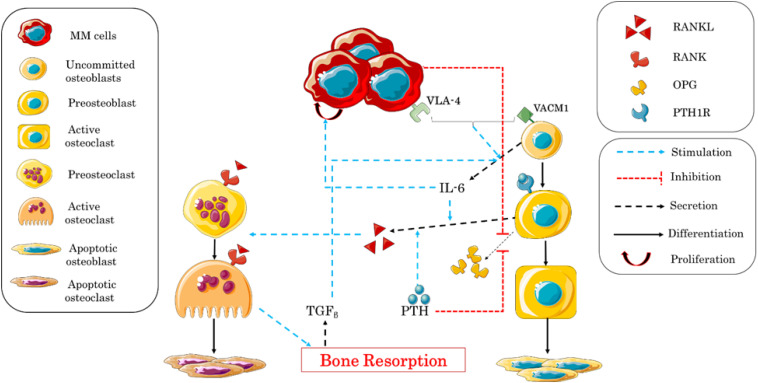
Schematic illustration of the biochemical interactions during the bone remodeling process after the adhesion of myeloma cells into the bone microenvironment. Interleukin-6 (IL-6) expression and multiple myeloma–bone marrow stromal cell (MM–BMSC) adhesion are the central factors controlling the process.

The interaction between MM and bone cells was also studied by Wang et al. (2011) based on Pivonka et al. (2008, 2010). The authors simplified the complex interactions between the two types of cells, in order to clarify the role of some biological factors involved in the remodeling process. The influence of IL-6 and the adhesion of MM–BMSC were the two main points addressed in this research (Table 4). The adhesion of myeloma cells is mediated by the adhesion molecule very-late antigen 4 (VLA-4), which binds to the vascular cell adhesion molecule 1 (VCAM-1) expressed by the uncommitted osteoblasts. In addition to VLA-4/VCAM-1 pathway, the authors considered the effects of TGF_β_ and PTH. For further clarifications, a description of the regulating mechanisms in MM–bone model is shown in Figure 3.

The obtained results were qualitatively and quantitatively consistent with the clinical observations, and the model provided a good and clear representation of MM–bone interactions during bone remodeling, which would allow to analyze the efficacy of some treatments.

**FIGURE 4:**
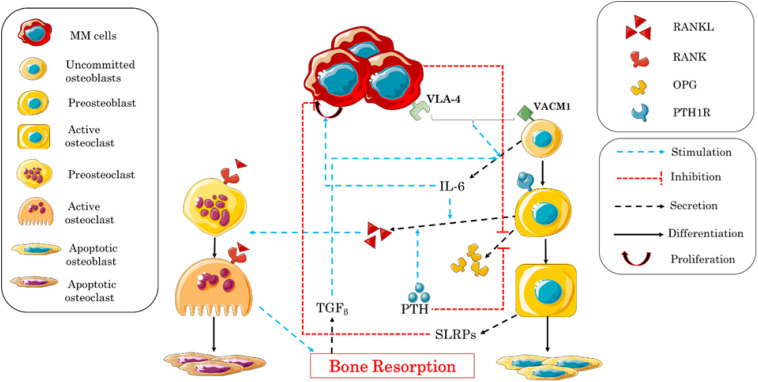
Schematic illustration of the biochemical interactions during the bone remodeling process after the adhesion of myeloma cells into the bone microenvironment. Interleukin-6 (IL-6) expression, multiple myeloma–bone marrow stromal cell (MM–BMSC) adhesion, and small leucine-rich proteoglycan (SLRP) expression are the central factors controlling the process.

In turn, Ji et al. (2014) constructed a model based on the work of Pivonka et al. (2008) to describe the behavior of bone cells and to explain bone degradation caused by myeloma. The study was divided into two parts. The first part described the factors stimulating the relationship between myeloma cells and the increase in the rate of bone resorption, and their effects on the proliferation of MM cells, similarly to the work of Wang et al. (2011). The second part described the relationship between the stimulation of the production of myeloma cells and the suppression of the activity of osteoblasts. The adhesion of MM–BMSC and the release or introduction of biochemical factors by MM were considered as blocking factors of the differentiation of BMSCs into mature and active osteoblasts. It should be noted that BMSCs stimulate the production of MM cells, while active osteoblasts increase the apoptosis of these cells (Matsumoto and Abe, 2011). In this study, the expression of VCAM-1 and the concentration of its receptor VLA-4 in the area are controlled by the concentration of MM, which is a function of IL-6 and VCAM-1 that activate their proliferation, and of small leucine-rich proteoglycan (SLRP) that represses their proliferation (Table 4). Figure 4 shows the different biochemical interactions involved in the development of MM-induced bone disease.

The authors considered many biochemical factors and attempted to create an exhaustive illustration of the mechanisms monitoring the process of the progression of the MM disease and its influence on bone remodeling. The results of their simulations were in concordance with the experimental data previously published, showing a fluctuation of the concentration of bone cells after the invasion of MM cells. Nevertheless, some biochemical factors involved in the process have been neglected, as the mechanism of MM interaction with bone cells is not completely clear. The effects of the treatments of this disease can later be incorporated using this model including a spatial dimension.

## Drug Interventions and Optimization

### Experimental Findings

Over time, several types of medical treatments have been developed to treat bone problems. Regardless of the disease’s type, the most widespread problem that bone suffers from is the loss of its mass. In the present subsection, we are going to present the main anti-resorptive therapies used to treat bone loss. First is estrogen, which plays an essential role in skeletal homeostasis in both women and men. It is used as so-called hormonal replacement therapy to prevent and treat osteoporosis, by increasing BMD at multiple sites. Indeed, estrogen has direct and indirect effects on osteoclasts’ formation, activity, and life span. The direct effect is presented in osteoclast possession of estrogen receptors (Oursler et al., 1991; Kousteni et al., 2001), which lead to osteoclast apoptosis (Kameda et al., 1997). Beyond this latter effect, estrogen blocks RANKL/M-CSF-induced activator protein-1-dependent transcription, which induces osteoclast differentiation suppression (Shevde et al., 2000). Besides, estrogen plays an indirect effect on osteoclastogenesis by blocking osteoblasts’ expression of RANKL and stimulating its expression of OPG (Hofbauer et al., 1999). Regardless of its remarkable advantages, estrogen has multiple side effects, namely, increasing BC risk and cardiovascular events (Rossouw et al., 2002).

Second, bisphosphonate, which is a powerful inhibitor of bone resorption and calcification (Russell, 2011), with a low effect on bone formation (Idris et al., 2008), is an extensively used drug to treat osteoporosis and PDB. In fact, prescribed doses of bisphosphonates can affect osteoclast recruitment, differentiation, and activity. Since bisphosphonate is incorporated into the bone matrix, it can disrupt osteoclasts’ ATP metabolic pathway during the resorption phase as it is captured by these resorbing cells during their functioning. Therefore, osteoclast activity is inhibited, and they undergo apoptosis (Arantes et al., 2010).

Teriparatide is another used drug, which is a truncated form of PTH. It induces an increase of bone formation accompanied by a smaller increase of bone resorption. Actually, continuous infusion of PTH causes anabolic and catabolic effects on the skeleton (Silva and Bilezikian, 2015). The catabolic action is illustrated by increasing an encoding gene of RANKL and inhibiting the OPG one, which leads to increase in RANKL/OPG ratio. Meanwhile, the anabolic action of PTH increases bone formation by stimulating osteoblasts transcription factors such as Runt-related transcription factor 2 (Runx2), osteocalcin, ALP, and collagen type 1 alpha1 (COL1A1) (Ogita et al., 2008). Based on these observations, teriparatide, which only represents the anabolic action of PTH, has been taken as an effective treatment drug of bone loss (Neer et al., 2001). Despite its good performance, this type of medicine may increase the risk of osteosarcoma (Vahle et al., 2004).

An important step in drug discovery was marked by the development of denosumab, which is a human monoclonal antibody to RANKL and acts as an anti-resorptive drug for osteoporosis and PDB. This drug mimics the effect of OPG in blocking RANKL–RANK binding. Actually, as well as OPG, denosumab binds RANKL with high affinity (Furman, 2007). Therefore, osteoclastogenesis is inhibited and bone resorption is reduced. Despite its capacity to inhibit excessive resorption, this type of drugs has also many side effects including back pain, pain in extremity, musculoskeletal pain, and hypercholesterolemia (Lewiecki, 2011).

### Mathematical Models: Mechanistic Pharmacokinetic/Pharmacodynamic Modeling

Intensive efforts have been made to develop mathematical models permitting the prediction of bone diseases and treatments’ effect on bone performance (Pivonka et al., 2012; Trichilo and Pivonka, 2017; Martin et al., 2020). Based on their results, they can suggest some therapeutic solutions for clinical uses. The latter purpose is reached generally using the pharmacokinetic/pharmacodynamic (PK/PD) modeling approach. Over the last years, the PK/PD modeling has been greatly developed in such a way the mechanism of the pathology, and the effect of the drug administration and dose on organs become clearer. Being interested in bone diseases and treatment, we are going to present some models that have investigated denosumab and PTH (1–34) drugs’ effect on bone remodeling. The other treatments such as bisphosphonates and estrogen have not been studied based on PK/PD models that consider bone remodeling mathematical formulations. The postmenopausal osteoporosis (PMO) category has been the most studied type of osteoporosis in these models. In the interest of determining the effect of denosumab on the remodeling process for postmenopausal women, Scheiner et al. (2014) have focused on investigating the biological factors triggering the PMO, which have been observed experimentally, and how the denosumab drugs could influence the biochemical interactions occurring. Based on the experimental data of Bekker et al. (2004), the authors have, first of all, created their PK model where the concentration of denosumab equation has been developed taking into consideration the absorption and degradation rates of the drug substance from the subcutaneous tissue to the blood serum. Thereafter, the PK model has been integrated into the mechanobiological bone remodeling model developed previously by Scheiner et al. (2013). In accordance with the experimental observations, PMO has been included into the model by increasing the RANKL/OPG’s concentrations’ ratio (Hofbauer and Schoppet, 2004). Indeed, the RANKL production has been increased by adding a parameter defining the PMO excess production of RANKL. Therefore, the RANKL concentration is adjusted and affects the differentiation of preosteoclasts into active osteoclasts. Besides, estrogen difficiency effect on bone mechanical sensitivity has been incorporated by decreasing the anabolic strength parameter and the parameter controling the level of RANKL production depending on the mechanical stimulus. Denosumab administration has been fixed after 6 months with different doses. The drug’s effect has been incorporated by modifying the activation function of preosteoclast differentiation by adding a term reflecting denosumab–RANKL binding in the calculation of RANKL concentration (Table 6). The outcomes have shown that there is an inhibition of preosteoclast differentiation and a temporary increase in bone volume formation after the period of denosumab administration. Otherwise, a higher dose of denosumab decreases bone turnover duration and leads to lengthening the volume fraction. The study’s results, which were compared with clinical experimental observations, demonstrated the model’s good capacity in mimicking quantitatively and qualitatively PMO bone disease as well as denosumab drug’s effect. Furthermore, the model was able to estimate the macroscopic bone stiffness, which could serve to assess the bone risk of fracture. However, the non-consideration of osteocytes and the large number of the model parameters are limitations that need to be exceeded in future researches.

**TABLE 6:** Methods adopted to incorporate denosumab and PTH drugs in mathematical models.

**Drug**	**Changed parameters**	**Method**	**References**
Denosumab	Activation function of preosteoclast differentiation	Adding a term reflecting denosumab–RANKL binding in the calculation of RANKL concentration.	Scheiner et al., 2014; Martínez-Reina and Pivonka, 2019
	Paracrine parameter of osteoblasts	The paracrine parameter depends on a function of denosumab concentration, this latter induce low RANKL effect on osteoclasts since the paracrine parameter is negative	Hambli et al., 2016
	Activation function of preosteoclast differentiation	RANK concentration is modified by adding denosumab occupancy term, which reflects denosumab–RANKL binding	Marathe et al., 2008
PTH	- Preosteoclasts proliferation term - Lining cells differentiation term - Osteoblast apoptosis term	- Preosteoclast proliferation and lining cell differentiation terms have been controlled by a function driven by the concentration of PTH and that depends on β-catenin concentration. - β-Catenin concentration depend on Dv1 and Axin–APC–GSK-3 complex - Axin–APC–GSK-3 complex production rate is regulated by PTH - The osteoblast apoptosis rate has been controlled by a function driven by the concentration of PTH and that depends on Bcl-2, CBEB, and Runx2 concentrations.	Trichilo et al., 2019
	Active osteoblast apoptosis rate	- The osteoblast apoptosis rate has been controlled by a function driven by the concentration of PTH and that depends on Bcl-2 concentration, which depends on PTH concentration.	Lavaill et al., 2020

*RANKL, receptor activator of NFκB ligand; PTH, parathyroid hormone.*

Recently, the treatment by denosumab for postmenopausal women has been further studied to analyze its long-term effects on the BMD values by developing a PK/PD model (Martínez-Reina and Pivonka, 2019). This study is based on the previously discussed study of Scheiner et al. (2014) and other studies (Martínez-Reina et al., 2008; Pivonka et al., 2008, 2010), where the RANK/RANKL/OPG pathway, TGF_β_, and the mineralization process of bone have been modeled. Denosumab’s effect on the remodeling process has been mediated by its effect on the RANK/RANKL/OPG pathway. Indeed, RANKL concentration involved in the remodeling process is adjusted by adding a mathematical term taking into account the denosumab–RANKL binding (Table 6), and no changes have been done on the production and degradation values. This way, the RANKL concentration will be reduced and the differentiation from preosteoclast to active osteoblast in PMO will, once again, be interrupted. On the other hand, the PMO disease has been also implemented in the bone remodeling mathematical model in a similar way to Scheiner et al.’s (2014) model. This study has provided very interesting results, as the model permitted to predict BMD increases in specific bone sites as shown in the experimental data of Bone et al. (2008) and have proved the importance of considering the bone mineralization process into the model as it influences the BMD and the BMD gains’ results as well.

In the work of Hambli et al. (2016), denosumab’s effect on the bone-specific region, which is the proximal femur of postmenopausal women, has been investigated. The authors were specifically interested in deducting the treatment’s dose and duration influence on the bone remodeling process by combining PK- and PD-based finite element (FE) models (PK/PD_FE). Based on the work of Scheiner et al. (2014), which was previously explained, the PK model has been established. Additionally to Scheiner et al.’s model, the authors have added a function to control the treatment dose administrated over time. Concerning the PD model, the authors have based their work on Komarova et al.’s (2003) bone remodeling mathematical model. In order to schematize denosumab’s effect, the authors have suggested a new formulation of the paracrine parameter related to osteoblasts. This choice has permitted to control RANK/RANKL/OPG system adding the denosumab serum concentration function in such a manner that the increase of the latter will induce a low effect of RANKL on osteoclasts, since the paracrine parameter is strictly negative. Thus, osteoclasts will be inhibited. We note that this model considered also the mechanical stimulus in the process and has been integrated into an FE model. Based on the FE model results, BMD values of proximal femur decreased by applying the mechanical loading without denosumab treatment, whereas denosumab intake twice a year during 3 years have shown a good consistency with an experimental study. Thus, the ability of this model to predict quantitatively and qualitatively BMU changes affected by denosumab treatment is confirmed.

Apart from the PMO, a study has addressed another type of problem inducing bone degradation (Marathe et al., 2008), which is MM. In this paper, the authors have coupled a bone homeostasis model to the denosumab PK model and pursued its effect on bone resorption by monitoring N-telopeptide (NTX) serum levels in MM patients. The PK model permits the calculation of the global concentration of the drug including the free and bonded ones to its receptors. Regarding the PD model, the authors suggested a differential equation determining NTX rate change depending on the free drug concentration. On the other hand, the bone homeostasis model, where the action of denosumab has been incorporated, has been based on Lemaire et al. (2004). Denosumab’s action was mediated by a change in the function controlling preosteoclast differentiation (Table 6). This change is represented by an alteration of RANK occupancy, which decreases due to the denosumab–RANKL binding term. The resulting decrease of osteoclast functioning is illustrated by a decrease in NTX serum concentration. The link between them has been depicted by an equation where NTX concentration is a function of active osteoclast concentration. This study demonstrated once again the importance of linking a PK model with a PD model to investigate a treatment effect on the progression of a certain disease.

As we have seen before, there are other treatments dedicated to stop bone loss. Apart from denosumab, which was considerably addressed, some researchers have investigated PTH action on bone remodeling using the PK/PD modeling. In the work of Trichilo et al. (2019), the PK model, as usual, has been devoted to define the treatment concentration change in the blood serum after leaving the subcutaneous tissue. In addition to the ODE describing the total concentration of PTH (1–34), which is a peptide fragment of the natural PTH, the authors have added an equation measuring the amount of drug that has already been absorbed and which depends on PTH serum concentration. Aiming to incorporate PTH drug anabolic effect on the bone remodeling of osteoporotic postmenopausal women, each of preosteoclast and active osteoblast concentration equations, described previously in Pivonka et al. (2008, 2013), Scheiner et al. (2013), and Pastrama et al. (2018) have been modified. Created active osteoblasts were subdivided into two equations, where the first represents the active osteoblasts, which derive from preosteoblast differentiation, while the second represents osteoclasts deriving from lining cells’ differentiation. Each of preosteoblast proliferation, lining cell differentiation, and even active osteoclast apoptosis were controlled by regulatory functions taking into account PTH effect. As we have seen in section “Experimental Findings,” PTH (1–34) acts on osteoblast transcription genes. Thus, the function controlling osteoblast apoptosis depended on Bcl-2 concentrations over time, while preosteoblast proliferation and lining cells’ differentiation were controlled by a function that depends on β-catenin. The model proposed has been successfully validated based on experimental data, and based on the results, the authors are estimating that the model would be very useful while studying rat models. However, computational modifications should be elaborated to translate the model to humans. In the same spirit, Lavaill et al. (2020) have addressed PTH treatment’s effect on PMO healing. However, this time, the authors have considered both anabolic and catabolic PTH actions according to the administration type. The PTH PK model was based on Trichilo et al. (2019), which has been calibrated according to the regular dose amount given to treat PMO. Some changes have been done on this model to characterize PTH effect. First, only an active osteoblast apoptosis rate has been adjusted by the effect of PTH (Table 6). Second, the term used for this adjustment, which represents the sigmoid function, depended only on Bcl-2 concentration. This anti-apoptotic molecule, in turn, depended on other substance concentration, notably Runx2 and phosphorylated cAMP response element-binding protein (pCREB), which drive the transcription rate of Bcl-2. This model results have also demonstrated the ability of the proposed formulations to replicate PMO and drugs’ effect on the remodeling process.

## Future Outlook and Conclusion

The skeletal system is mainly made up of bone tissue. Thus, to maintain its multiple functions, bone needs to be constantly renewed. The current paper represents a description of bone remodeling process in healthy and pathological conditions, and the importance of the biological and biochemical interactions in influencing bone quality was highlighted. For this reason, the mathematical model developed in this area of research has been devoted to the biological conditions. Through the present review, we came up with these summary points:

1. Local and systemic factors are mandatory for the identification of bone tissue changes.
2. An integrative view of genes and signaling pathways that control the bone physiology and pathophysiology is mandatorily needed to develop mathematical models.
3. The models developed need to converge toward a general formulation permitting to study any patient case.
4. The high number of parameters used in each mathematical model is making these more complex and increases the results’ margin of error.
5. Each tumor cell type is distinguished by the involvement of typical biological factors (e.g., DKK-1, Wnt, and PSA in PC and IL-6 plus VLA-4/VCAM-1 pathway in MM).
6. Major advances in the diagnosis and treatment of bone diseases have been achieved, but many methods still need investigation by means of PK/PD models.
7. Although being one of the major causes inducing osteoporosis development, BC has not been described using mathematical models to investigate its effect on bone remodeling, and the other types of cancer still require massive investigations for a better understanding of their influence on the interactions between bone cells.

By virtue of their serious investigations of cancer–bone interaction, researchers discovered very relevant findings, which generally reflect the experiment or the clinical observations. Nevertheless, it is always possible to improve the proposed models by investigating more biological factors, constructing 1D or 2D models representing the evolution of the BMU of a damaged bone or studying patient-specific models, which can take into consideration other parameters (e.g., age, sex, and cancer stage). These improvements will allow to test the efficacy of therapeutic methods used for all cancers relatively to bone diseases. Besides, they will make our FE analysis more accurate while studying the remodeling effect on the bone geometry and its mechanical performance.

## Author Contributions

IA wrote this review article under the guidance and direction of these supervisors AB and PC who corrected and participated in the structuring and discussion of this article. All authors contributed to the article and approved the submitted version.

## Conflict of Interest

The authors declare that the research was conducted in the absence of any commercial or financial relationships that could be construed as a potential conflict of interest.
